# Meetings of Societies

**Published:** 1913-06

**Authors:** 


### MEETINGS OF SOCIETIES.

### Bristol Medico-Chirurgical Society.

*March 14th*, 1913.

Dr. WALTER C. SWAYNE, President, in the Chair.

Dr. P. WATSON-WILLIAMS showed a case, one year after removal, of an **Extensive epithelioma of the larynx** without recurrence, but with some stridor from cicatricial contraction.

Mr. HUBERT CHITTY showed an infant, aged six weeks, with on the left side **Congenital absence of the ulna and two digits with their metacarpals,** and on the right side absence of **one digit and its metacarpal.**

Dr. CAREY COOMBS, in a paper on **Certain adventitious pericardiac sounds,** pointed out the frequent occurrence in cases of cardiac valvular lesions of pulmonary crepitations over the third left interspace in front, particularly in cases of mitral stenosis. He said that these were probably due to collapse of the lung margin, owing to pressure of the enlarged conus arteriosus, together with hyperæmia, and were of interest in that they not uncommonly led to a mistaken diagnosis of pulmonary tuberculosis. The stationary condition of the signs, together with the absence of tubercle bacilli in the sputum, were sufficient to negative the presence of tuberculosis.—Dr. J. MICHELL CLARKE remarked that the limitation of these crepitations to the first deep inspiration during auscultation pointed to their being due to a condition of atelectasis.—Dr. J. R. CHARLES, in an examination of some thousands of post-mortem records, had found that the coexistence of pulmonary tuberculosis and mitral stenosis was a rare occurrence.

Dr. Charles read a paper on the **Etiology of migraine,** in which he propounded the theory that the symptoms of migraine were due to a transient swelling of the pituitary gland. Such swelling was known during pregnancy, and from the anatomical position of the gland it was conceivable that if swollen it might produce effects on the optic nerves and those in the walls of the cavernous sinus. The vasomotor effects might be explained by an increased secretion from the posterior lobe.—Mr. H. Elwin Harris did not think that the theory could explain in his own case the occurrence of hemianopsia half an hour before the headache.—Dr. Coombs did not see how the association of migraine and epilepsy could be explained by this theory.

Dr. Watson-Williams read notes and showed a specimen from a case of **Cerebro-spinal rhinorrhœa,** in which there was an opening found in the dura and bone of the anterior fossa post-mortem, death occurring from meningitis from a complicating frontal sinusitis. During life the cerebro-spinal fluid had caused a fluctuating swelling at the inner orbital margin, thus simulating an ethmoidal mucocele.

---

*April 9th*, 1913.

Dr. Walter C. Swayne, President, in the Chair.

Mr. E. H. E. Stack, in a demonstration on some varieties of **Arm splints,** advocated the use of a malleable metal anterior angular splint for the treatment of fractures of the forearm. For all injuries about the elbow-joint he had found the position of acute flexion to give the best results, and demonstrated a simple method for putting up an arm in that position. He advocated the early use of active rather than passive movements, the patient being better able to judge the amount of movement possible without risk of damage.

Mr. Charles Corfield, read a paper entitled **"What is an Accident?"** (*vide* p. 148).

Mr. W. Stuart V. Stock related two cases in which death occurred two or three days after an operation from **Heart failure** in patients the subject of fatty heart, although they had shown no bad signs during a prolonged administration of an anæsthetic.

Mr. A. Rendle Short, in a paper on the **Nature of surgical shock,** pointed out that the two great objections to the generally-accepted theory of Crile and Mummery—that it was due to vaso-motor centre fatigue—were that the peripheral vessels were found to be constricted and not dilated, and also that the centre could be shown experimentally not to be exhausted. He had examined the venous blood of a number of patients the subject of shock, but had been unable to confirm the theory of Yandel

Henderson that shock was due to a deficiency of $CO_2$ in the blood. His own view was that the symptoms were due to a loss of fluid from the blood which apparently passed out into the tissues and was possibly squeezed out by vasomotor reflexes.

---

*May 14th,* 1913.

Dr. WALTER C. SWAYNE, President, in the Chair.

Dr. CAREY COOMBS showed a case of **Congenital Cardiac lesion.** The patient, a boy of four, had been first noticed to get blue at times when six months old. He had clubbed fingers and cyanosis, and was undergrown. The cyanosis was paroxysmal, and induced by exertion and with colds. The heart was enlarged to right and to left, and had a loud rasping bruit. The speaker gave reasons for assuming the lesions to be congenital pulmonary stenosis, coupled with patency of the interventricular septum. He considered the prognosis to be bad.—Dr. EVERY CLAYTON thought the boy had adenoids, and that oxygenation would be improved if they could be safely removed.

Dr. E. C. WILLIAMS showed three cases: (1) **Ataxia** in child of three. The child could only stand if supported. The gait on attempting to walk was characteristically ataxic. The knee jerks were exaggerated. The plantar reflexes flexor. No optic atrophy or neuritis was present. The condition seemed stationary, not advancing as in a Friedrich's ataxy. (2) A case of the **Juvenile type of muscular atrophy.** The boy was eight years old. His health had been good until two years of age. He began then to fall easily, especially if tired. During the last six weeks he had shewn some hypertrophy of the calves. He had not considered the case to be one of pseudohypertrophic paralysis. (3) A case of hepatic enlargement with ascites, and a purpuric eruption. Illness began with abdominal pain and hæmatemesis. Large quantities of fluid had been withdrawn from the abdomen repeatedly. There appeared to be portal obstruction.—Dr. PARKER thought the duration of the trouble was greater than that in cases of Henoch's purpura.—Dr. MICHELL CLARKE suggested a diagnosis of congenital syphilis of the liver.—Dr. EVERY CLAYTON said he had recently seen a case of purpura, the purpuric spots coming out in successive crops, which had lasted six months. Dr. STACK thought granular kidney might be the cause.—Dr. C. WILLIAMS replied that he had purposely named this case the juvenile type of muscular atrophy, as the case was of long standing (two years). The muscular weakness was rapidly advancing, and in a typical case of pseudohypertrophic paralysis there would be well-marked enlargement of the buttocks. There is no essential difference between the two diseases; they are probably only modifications of the same disease, so there can be no possible reason in calling the case pseudohypertrophic, when the only pseudohypertrophy which exists is that found in the juvenile type of muscular atrophy.

Dr. D. A. ALEXANDER showed a case of **Brachial hypertrophy.** The youth had massive upper limbs and slight enlargement of the right leg, but a small head. The suggestion was made that the boy's condition illustrated reversion to an ancestral, less developed type of human being.—Dr. CLARKE thought there might be some pituitary gland change causing the hypertrophy.

J. LACY FIRTH.

A. J. WRIGHT, *Hon. Sec.*

